# Trace mineral concentrations and accretion rates in the empty body and body tissues of growing Fleckvieh (German Simmental) bulls

**DOI:** 10.5194/aab-66-265-2023

**Published:** 2023-09-19

**Authors:** Aniela C. Honig, Vivienne Inhuber, Hubert Spiekers, Wilhelm Windisch, Kay-Uwe Götz, Gerhard Strauß, Thomas Ettle

**Affiliations:** 1 Bavarian State Research Center for Agriculture, Institute for Animal Nutrition and Feed Management, Prof.-Duerrwaechter-Platz 3, 85586 Poing, Germany; 2 Chair of Animal Nutrition, Technical University of Munich, Liesel-Beckmann-Strasse 2, 85354 Freising, Germany; 3 Bavarian State Research Center for Agriculture, Institute for Animal Breeding, Prof.-Duerrwaechter-Platz 1, 85586 Poing, Germany; 4 Bavarian State Research Center for Agriculture, Department of Laboratory Analytics, Lange Point 4, 85354 Freising, Germany

## Abstract

This research project aimed to generate basic data for specifying the trace mineral requirements of Fleckvieh (German Simmental) bulls. Hence, the concentrations of the trace minerals iron (Fe), zinc (Zn), copper (Cu), and manganese (Mn) in the empty-body and body tissue fractions of growing Fleckvieh bulls slaughtered at 120–780 kg live weight were determined. Results were used to calculate trace mineral accretion rates. Fe and Zn represented the largest shares in the animals' bodies. The Zn accretion increased, while Mn accretion steadily declined during cattle growth. Fe accretion attained a maximum at 400 kg live weight. Cu accretion declined until 600 kg live weight and then increased slightly afterwards. The provided data may be used to adjust the recommendations with respect to the trace mineral requirements of growing Fleckvieh bulls.

niela.honig@lfl.bayern.de]Aniela C.Honig homas.ettle@lfl.bayern.de]ThomasEttle

## 1 Introduction

In cattle, trace minerals comprise less than 50 mg per kilogram body weight (Kirchgeßner, 2004). Despite their low content, adequate trace mineral nutrition is essential to maintain physiological body functions and ensure animal growth. A deficient trace mineral supply results in various symptoms. Iron (Fe) deficiency entails anemia; this commonly occurs in calves, which have high iron requirements for physiological growth but must cope with the low iron concentration in milk (Wysocka et al., 2020). Mohri et al. (2010) demonstrated that an additional administration of iron to newborn calves promoted their growth. Impaired animal growth, skin lesions, delayed wound healing, hair and coat impairment, diarrhea, and increased susceptibility to infections are indicators of zinc (Zn) deficiency (Machen et al., 1996). Regarding copper (Cu) supply, Abramowicz et al. (2021) reported Cu deficiency to result in anemia due to reduced red blood cell count and hemoglobin concentration in dairy cattle. Furthermore, the authors found a reduction in lymphocytes and neutrophils, which was also associated with Cu deficiency and an impaired immune function by Cerone et al. (1998). A manganese (Mn) shortage in cattle manifests itself in skeletal malformation and reduced growth (Staley et al., 1994). Trace mineral malnutrition and associated deficiency symptoms can occur when animals are not fed according to their specific requirements, and this should be avoided to ensure good livestock performance and welfare.

An important step in calculating the trace mineral requirements for growing cattle within the factorial requirement calculation method is to determine the animals' trace mineral accretion at various stages of growth. Hence, this research project examines trace mineral concentrations and accretion rates in the empty body (the animals' complete body minus the contents of the urinary bladder and gastrointestinal tract) and individual body tissues of growing Fleckvieh (German Simmental) bulls. The Fleckvieh breed is a common dual-purpose cattle breed in southern Germany and Austria and simultaneously provides high milk (8080 kg yr ${}^{-1}$ ; Schäffer, 2021) and meat (57 % dressing percentage; Schäffer, 2022) yields. The breed's performance potential has been improved by selective breeding and progress in cattle farming and feeding during the past decades. In practice, this is reflected in increased daily weight gains and final animal weights, which may be associated with changes in the animals' energy and nutrient requirements. Basic research into energy and nutrient accretion in growing Fleckvieh bulls was performed almost 3 decades ago, revealing respective protein and energy accretion rates of 208 g d ${}^{-1}$ and 12.6 MJ d ${}^{-1}$ in 200–650 kg bulls (Kirchgessner et al., 1994a, b; Schwarz et al., 1995). Recently, Honig et al. (2022a) showed that macromineral accretion rates in Fleckvieh bulls used in this work differ from those reported by Schwarz et al. (1995). Hence, the following two related questions arise:

- Has trace mineral accretion also changed to an equivalent degree during the past decades?
- Should feeding recommendations be adjusted to the requirements of today's growing bulls?

This research project aimed to determine the content and accretion of the trace minerals iron, zinc, copper, and manganese in the empty-body and body tissue fractions of growing Fleckvieh bulls.

## 2 Material and methods

### 2.1 Animals and treatments

The research was conducted at the Bavarian State Research Center for Agriculture (LfL), according to the European guidelines for animal experiments (Directive 2010/63/EU, 2010), and approved by the LfL Committee for Ethics of Animal Experiments. Material and methods employed during calf rearing and bull fattening have been previously described by Honig et al. (2020).

The trial included 72 male Fleckvieh calves (German Simmental; aged 42 d ± 9 with a body weight (BW) of 80 kg ± 6), randomly acquired from cattle farms in Bavaria, southern Germany. The calves were fed with restricted amounts of milk replacer (120 g L ${}^{-1}$ ), with a maximum of 6 L d ${}^{-1}$ , and a total mixed ration (TMR) based on concentrates and hay until weaning at an average BW of 121 kg ± 10 (age of 86 d ± 9). Subsequently, the animals were fed a TMR based on maize silage and concentrates for ad libitum intake. During the rearing phase, the feed intake of each animal group was recorded daily, and individual milk replacer intake was recorded using automatic calf feeders. The calves' BW was determined using a calf scale every 14 d.

The fattening period was initiated at an average BW of 225 kg ± 29 and age of 154 d ± 15. At this stage, the bulls were randomly allocated to normal-energy (NE) and high-energy (HE) treatment groups that were fed rations with 11.6 and 12.4 MJ ME kg ${}^{-1}$ dry matter (DM) for ad libitum intake, respectively. Differences in TMRs' energy concentrations were achieved by varying the amount of maize silage and concentrates in the rations. The feed mineral content was based on the recommended mineral supply for fattening bulls (GfE, 1995). The feed trace mineral concentrations were kept constant in relation to feed DM. Hence, the feed trace mineral content did not differ between the NE and HE groups. During the fattening period, the individual feed intake was recorded daily, and BW was determined using a cattle scale at 4-week intervals.

### 2.2 Slaughter and body tissue sampling

Animals were slaughtered at the LfL research abattoir in Grub, Germany, in compliance with Council Regulation (EC) No. 1099/2009 (2009). Slaughter and tissue-sampling methods have been previously described by Honig et al. (2020, 2022a, b). In short, bulls from both feeding groups were slaughtered at final live weights of 120, 200, 400, 600, and 780 kg. During slaughter, the bulls' empty-body weights (EBWs) were determined as the final live weight minus the contents of the urinary bladder and gastrointestinal tract (GIT). Subsequently, the entire empty body was dissected to individual body tissue fractions (hide, blood, organs, empty GIT, body fat, muscle, tendon, and bone), which were then homogenized, sampled, and analyzed for their trace mineral concentration. The experimental design allowed for the analysis of the empty-body and individual-body-tissue trace mineral compositions at different stages of the animals' maturity. Feeding varying energy concentrations reflected the range of different growth intensities under practical conditions.

### 2.3 Trace mineral analyses of feedstuffs and body tissues

The trace mineral analyses were conducted at the LfL Department of Laboratory Analytics according to the methods of the Association of German Agricultural Analytic and Research Institutes (VDLUFA, 2012). Three different samples of individual feedstuffs and body tissues were analyzed for their Fe, Zn, Cu, and Mn contents. For this, feedstuff and body tissue samples were homogenized and dissolved in nitric acid and hydrogen peroxide. Trace minerals were subsequently extracted by pressure digestion, using a microwave pressure digestion system (MARS 6 240/50, CEM Corporation, Matthews, USA) and analyzed using inductively coupled plasma optical emission spectrometry (Agilent 725 ICP-OES, Agilent Technologies, Santa Clara, USA). Applying a daily five-point calibration method based on certified standard solutions (Merck KGaA, Darmstadt, Germany) ensured good analysis quality. Analytical results were verified through reference materials and control samples.

The trace mineral concentrations of feedstuffs and TMRs given during calf rearing and the fattening period are presented in Table 1 and were calculated based on the TMRs' compositions and the mineral content in the individual feed components. The bulls' empty-body mineral contents were calculated based on their body tissue composition (Honig et al., 2022b) and the mineral contents in the individual body tissues.

**Table 1:** Trace mineral concentrations in feedstuffs and total mixed rations (TMRs) fed during calf rearing and the fattening period.

Feedstuffs and TMRs	Minerals			
	Iron	Zinc	Copper	Manganese
	(mg per kg DM)	(mg per kg DM)	(mg per kg DM)	(mg per kg DM)
Barley	52	32	6	14
Brewer's yeast	181	76	8	32
Calcium carbonate, cattle salt	0	0	0	0
Calf milk replacer	99	96	5	46
Feed-grade urea, 46.5% N	0	0	0	0
Hay	182	40	9	54
Maize grain	34	20	4	4
Maize silage	70	24	7	21
Minerals: 26 % Ca and 2 % P	1455	7954	895	4586
Molasses	6	13	1	61
Pressed beet pulp	1033	22	5	107
Rapeseed meal	197	85	8	77
Soybean oil	0	0	0	0
Wheat	49	33	7	38
Normal-energy TMR	110	98	14	67
High-energy TMR	144	101	14	70

The normal-energy TMR contains 80 % maize silage and 20 % concentrates (% DM), resulting in 11.6 MJ ME kg ${}^{-1}$ DM (where ME represents metabolizable energy). The high-energy TMR contains 40 % maize silage and 60 % concentrates (% DM), resulting in 12.4 MJ ME kg ${}^{-1}$ DM.

### 2.4 Data analysis

Statistical analysis of feed and body mineral contents was performed using the PROC MIXED procedure of SAS (Version 9.4, SAS Institute, Cary, NC, USA) and the Kenward–Roger method to provide corrected degrees of freedom. The analysis included a two-way analysis of variance (ANOVA) with interaction (feed energy, weight group, feed energy × weight group). Differences between the groups were tested using the PDIFF option with effects stated as significant when p<0.05 . The results are presented as least-squares means (LSMs) and the standard error of the mean (SEM). One animal with a 780 kg live weight showed a Mn content in the GIT that exceeded the weight group average by more than 2 standard deviations and, thus, had to be excluded from the Mn statistical analysis.

Results on empty-body and body tissue trace mineral concentrations were compared to recalculated data by Kirchgessner et al. (1994b) of Fleckvieh bulls with 200–650 kg live weight fed ad libitum. For this purpose, a relation of the empty-body weight to live weight of 0.88 for 200 kg bulls and of 0.93 for 400–780 kg bulls was assumed as inferred from the present study.

To calculate the bulls' mineral accretion, third-order polynomial regression equations and their derivatives were used according to methods described by Honig et al. (2022b). The regression analyses were calculated using the PROC NLIN procedure in SAS and based on Eq. (1):

1 $$y_{i}=a{\mathrm{LW}}_{i}+b{{\mathrm{LW}}_{i}}^{2}+c{{\mathrm{LW}}_{i}}^{3}+\;e_{i},$$

where LW is the live weight and e is the residual error.

The residuals of the fitted models for the NE and HE bulls were calculated to estimate significant differences between the feed intake groups and to evaluate the goodness of fit of the regression equations. A two-way ANOVA (interaction feed energy × weight group) showed no significant differences in the residuals of both feed intake groups. Hence, combined regression equations were calculated for both groups and presented in the results. The model predictive performance was determined by calculating the coefficient of determination ( $R^{2}$ ) for each equation as follows: $R^{2}$ = 1 - SSE / CSS, where SSE is the sum of squares error and CSS is the corrected sum of squares.

## 3 Results and discussion

### 3.1 Fattening performance and trace mineral intake

The results on the feed intake, fattening performance, and efficiency of growing Fleckvieh beef bulls have been published by Honig et al. (2020, 2022b). In brief, bulls in the NE and HE treatment groups exhibited respective daily weight gains of 1699 and 1792 g d ${}^{-1}$ from 200 to 780 kg live weight ( p<0.1 ). HE feeding significantly increased daily weight gains at particular stages of the fattening period and, thus, shortened the fattening period in 780 kg HE-fed bulls by 21 d ( p<0.05 ). Increasing the amount of concentrate in the HE ration led to significantly greater daily DM (Honig et al., 2020) and trace mineral intake in HE bulls (Table 2). The trace mineral concentrations in NE and HE rations covered the bulls' requirements as specified by GfE (1995), preventing the risk of trace mineral malnutrition and associated deficiency symptoms.

**Table 2:** Trace mineral intake of bulls in normal-energy (NE) and high-energy (HE) treatment groups in different weight ranges.

Mineral intake	Weight range								SEM	p value		
	80–120 kg	120–200 kg	200–400 kg		400–600 kg		600–780 kg			feed	weight	feed ×
												weight
	n = 72	n = 64	NE	HE	NE	HE	NE	HE				
			n = 27	n = 27	n = 18	n = 18	n = 9	n = 9				
Iron (mg d ${}^{-1}$ )	385	856	856 ${}^{A}$	1235 ${}^{B}$	1107 ${}^{A}$	1701 ${}^{B}$	1327 ${}^{A}$	1857 ${}^{B}$	27.88	<0.0001	<0.0001	<0.0001
Zinc (mg d ${}^{-1}$ )	317	748	748 ${}^{A}$	950 ${}^{B}$	1011 ${}^{A}$	1286 ${}^{B}$	1133 ${}^{A}$	1382 ${}^{B}$	21.32	<0.0001	<0.0001	<0.0001
Copper (mg d ${}^{-1}$ )	40	95	107 ${}^{A}$	134 ${}^{B}$	145 ${}^{A}$	189 ${}^{B}$	162 ${}^{A}$	194 ${}^{B}$	3.23	<0.0001	<0.0001	<0.0001
Manganese (mg d ${}^{-1}$ )	224	527	535 ${}^{A}$	687 ${}^{B}$	730 ${}^{A}$	934 ${}^{B}$	838 ${}^{A}$	990 ${}^{B}$	15.58	<0.0001	<0.0001	<0.0001

Means within a weight range sharing the same superscript are not significantly different.

### 3.2 Empty-body trace mineral composition

Empty-body weights (EBWs) of the 120, 200, 400, 600, and 780 kg weight groups were 104, 176, 370, 553, and 734 kg, respectively (Honig et al., 2022b). The average empty-body and body tissue trace mineral compositions in bulls in different weight groups are presented in Table 3. The trace mineral concentrations in Table 3 refer to natural body tissue including moisture. As the dietary energy concentration had no significant effect on the body trace element concentration of bulls in the NE and HE treatment groups, the mean values for both the NE and HE groups are shown. Results were compared to recalculated empty-body trace mineral concentrations in Fleckvieh bulls used in prior work by Kirchgessner et al. (1994b) that were fed ad libitum at defined live weights. The authors described the average empty-body and carcass trace mineral concentration in 54 Fleckvieh bulls with a 200–650 kg live weight. Therefore, the body tissues and parts (muscle, tendon, bone, fat, hide, organs and GIT, and head and feet) were ashed (except for fat tissue, which was analyzed as fresh matter) and afterwards analyzed using a spectral photometer. Hence, limitations in comparability are caused by dissimilarities in tissue collection during slaughter and carcass processing and different sample preparation and analysis methods.

**Table 3:** Empty-body and body tissue trace mineral concentrations in bulls in different weight groups (natural tissue including moisture).

Body fractions	Mineral composition	Weight group					SEM	p value
		120 kg	200 kg	400 kg	600 kg	780 kg		weight
		n = 8	n = 10	n = 18	n = 18	n = 18		
Empty body	Iron (mg kg ${}^{-1}$ )	38.7 ${}^{\mathrm{AB}}$	35.8 ${}^{A}$	37.3 ${}^{A}$	40.9 ${}^{B}$	38.7 ${}^{\mathrm{AB}}$	0.51	0.0213
	Zinc (mg kg ${}^{-1}$ )	27.8 ${}^{A}$	25.3 ${}^{B}$	27.2 ${}^{A}$	30.6 ${}^{C}$	31.4 ${}^{C}$	0.36	<0.0001
	Copper (mg kg ${}^{-1}$ )	3.7 ${}^{A}$	3.8 ${}^{A}$	2.9 ${}^{B}$	2.5 ${}^{C}$	2.2 ${}^{C}$	0.09	< 0.0001
	Manganese (mg kg ${}^{-1}$ )	2.50 ${}^{A}$	2.22 ${}^{\mathrm{AB}}$	1.93 ${}^{B}$	1.35 ${}^{C}$	1.17 ${}^{C}$	0.10	<0.0001
Hide	Iron (mg kg ${}^{-1}$ )	23.4 ${}^{A}$	17.6 ${}^{B}$	15.9 ${}^{B}$	11.0 ${}^{C}$	11.6 ${}^{C}$	0.70	<0.0001
	Zinc (mg kg ${}^{-1}$ )	15.6 ${}^{A}$	11.1 ${}^{B}$	9.6 ${}^{B}$	9.6 ${}^{B}$	10.0 ${}^{B}$	0.34	<0.0001
	Copper (mg kg ${}^{-1}$ )	1.4 ${}^{\mathrm{AB}}$	1.4 ${}^{A}$	1.2 ${}^{\mathrm{BD}}$	0.7 ${}^{C}$	1.1 ${}^{D}$	0.04	<0.0001
	Manganese (mg kg ${}^{-1}$ )	0.88 ${}^{A}$	0.88 ${}^{A}$	0.65 ${}^{B}$	0.40 ${}^{C}$	0.43 ${}^{C}$	0.04	<0.0001
Blood and organs	Iron (mg kg ${}^{-1}$ )	139.3 ${}^{A}$	141.0 ${}^{A}$	169.8 ${}^{B}$	232.2 ${}^{C}$	235.1 ${}^{C}$	5.95	<0.0001
	Zinc (mg kg ${}^{-1}$ )	15.0	14.5	15.6	15.5	16.3	0.22	0.1363
	Copper (mg kg ${}^{-1}$ )	20.3 ${}^{A}$	20.9 ${}^{A}$	17.0 ${}^{B}$	14.0 ${}^{C}$	12.9 ${}^{C}$	0.55	<0.0001
	Manganese (mg kg ${}^{-1}$ )	0.48 ${}^{\mathrm{AC}}$	0.56 ${}^{\mathrm{AB}}$	0.57 ${}^{B}$	0.48 ${}^{C}$	0.48 ${}^{C}$	0.01	0.0067
Gastrointestinal tract	Iron (mg kg ${}^{-1}$ )	47.4 ${}^{A}$	72.7 ${}^{\mathrm{AB}}$	79.5 ${}^{B}$	67.8 ${}^{\mathrm{AB}}$	52.9 ${}^{A}$	3.35	0.0169
	Zinc (mg kg ${}^{-1}$ )	23.8	21.5	23.0	21.8	22.4	0.41	0.4915
	Copper (mg kg ${}^{-1}$ )	1.7	1.9	1.7	1.8	1.8	0.04	0.5012
	Manganese (mg kg ${}^{-1}$ )	30.00	28.36	30.81	27.32	26.83	1.48	0.8468
Fat tissue	Iron (mg kg ${}^{-1}$ )	12.4 ${}^{A}$	7.9 ${}^{B}$	5.8 ${}^{C}$	4.7 ${}^{D}$	4.6 ${}^{D}$	0.33	<0.0001
	Zinc (mg kg ${}^{-1}$ )	7.9 ${}^{A}$	5.1 ${}^{B}$	4.2 ${}^{C}$	3.9 ${}^{\mathrm{CD}}$	3.7 ${}^{D}$	0.17	<0.0001
	Copper (mg kg ${}^{-1}$ )	1.1 ${}^{\mathrm{AB}}$	1.3 ${}^{A}$	0.9 ${}^{B}$	1.1 ${}^{B}$	1.0 ${}^{B}$	0.04	0.0242
	Manganese (mg kg ${}^{-1}$ )	0.28 ${}^{A}$	0.09 ${}^{B}$	0.07 ${}^{\mathrm{BC}}$	0.05 ${}^{C}$	0.06 ${}^{C}$	0.01	<0.0001
Muscle tissue	Iron (mg kg ${}^{-1}$ )	11.3 ${}^{A}$	12.6 ${}^{A}$	15.6 ${}^{B}$	18.4 ${}^{C}$	21.6 ${}^{D}$	0.47	<0.0001
	Zinc (mg kg ${}^{-1}$ )	30.0 ${}^{A}$	31.1 ${}^{A}$	40.6 ${}^{B}$	49.2 ${}^{C}$	52.4 ${}^{D}$	1.08	<0.0001
	Copper (mg kg ${}^{-1}$ )	1.3	1.5	1.3	1.4	1.3	0.04	0.3772
	Manganese (mg kg ${}^{-1}$ )	0.10	0.10	0.09	0.10	0.09	0.001	0.5171
Tendon	Iron (mg kg ${}^{-1}$ )	10.0	8.2	8.8	9.6	9.7	0.23	0.1438
	Zinc (mg kg ${}^{-1}$ )	10.1 ${}^{A}$	10.0 ${}^{A}$	10.6 ${}^{A}$	12.8 ${}^{B}$	12.4 ${}^{B}$	0.27	0.0004
	Copper (mg kg ${}^{-1}$ )	1.0	1.2	1.0	1.1	1.1	0.04	0.6049
	Manganese (mg kg ${}^{-1}$ )	0.11 ${}^{A}$	0.09 ${}^{\mathrm{AB}}$	0.08 ${}^{B}$	0.08 ${}^{B}$	0.08 ${}^{B}$	0.003	0.0373
Bone	Iron (mg kg ${}^{-1}$ )	50.4 ${}^{A}$	35.8 ${}^{B}$	33.5 ${}^{B}$	29.8 ${}^{\mathrm{BC}}$	26.0 ${}^{C}$	1.44	<0.0001
	Zinc (mg kg ${}^{-1}$ )	49.5 ${}^{A}$	45.0 ${}^{\mathrm{AC}}$	37.1 ${}^{B}$	40.9 ${}^{\mathrm{BCD}}$	45.1 ${}^{\mathrm{AD}}$	0.88	0.0002
	Copper (mg kg ${}^{-1}$ )	1.3 ${}^{A}$	1.4 ${}^{A}$	0.8 ${}^{B}$	0.8 ${}^{B}$	0.7 ${}^{B}$	0.07	0.0008
	Manganese (mg kg ${}^{-1}$ )	0.75 ${}^{A}$	0.41 ${}^{B}$	0.21 ${}^{B}$	0.24 ${}^{B}$	0.20 ${}^{B}$	0.04	0.0003

Means within a row sharing the same superscript are not significantly different.

The bulls' empty-body trace mineral concentration changed during animal growth. Growing bulls showed increasing Zn but decreasing Cu and Mn concentrations. The Fe concentration in the empty body was comparable in the lowest and highest weight groups. Comparing the results to recalculated empty-body trace mineral compositions showed lower Fe, Cu, and Mn concentrations but higher Zn concentrations in Fleckvieh bulls used in previous work (Kirchgessner et al., 1994b). The most notable difference occurred in bulls with a 200 kg live weight used in this work, in which Cu and Mn concentrations were more than twice as high as in bulls used in former studies.

Increasing amounts of Zn in the empty body resulted from the high Zn concentration in the animals' muscle tissue. Fleckvieh bulls feature high proportions of muscle tissue, even at high live weights (Honig et al., 2022b). Muscle Zn and Fe concentrations increased by 75 % and 91 % from the lowest to the highest weight group, respectively, while Cu and Mn concentrations did not change during growth. This observation confirms research by Kirchgessner et al. (1994b), who reported increasing Zn and Fe concentrations but constant Cu and Mn concentrations in muscle tissue of growing bulls fed ad libitum. However, the muscle Cu concentration in Fleckvieh bulls used in this work was twice as high as in bulls used in prior studies, which featured an average muscle Cu concentration of 0.6 mg per kilogram of tissue (Kirchgessner et al., 1994b).

Growth-associated changes in muscle mineral concentration were also observed by Giuffrida-Mendoza et al. (2007). Furthermore, the authors reported differences in muscle P and Fe concentrations in buffalo and cattle species (Giuffrida-Mendoza et al., 2007). Breed and muscle type can also affect the muscle trace mineral concentration (Cabrera et al., 2010; Ramos et al., 2012; Somogyi et al., 2015; Domaradzki et al., 2016). Moreover, muscle mineral concentrations differ in different sexes within the same breed (Mateescu et al., 2013). Species-, breed-, sex-, and muscle-type-dependent differences in muscle mineral concentrations may be a result of differing intramuscular fat contents.

The highest tissue Fe concentration was observed in the blood and organs fraction, where the Fe concentration increased by 69 % during growth. Furthermore, blood and organ tissue showed the highest tissue Cu concentration, whereas the lowest Cu concentration was observed in bone tissue of 780 kg bulls. The Fe, Cu, and Mn concentrations in bone tissue decreased from the lowest to the highest weight group, which can be attributed to increasing bone mineralization. Former research has indicated decreasing Fe but constant Zn, Cu, and Mn concentrations in the bone tissue of growing bulls (Kirchgessner et al., 1994b).

Concentrations of Fe, Zn, and Mn in fat tissue decreased during animal growth as a result of increasing crude fat proportions. This observation partly confirms research by Kirchgessner et al. (1994b), who reported that Fe and Zn concentrations decreased from 12.8 to 4.7 and from 7.1 to 5.3 mg per kilogram of fat tissue, respectively, while Cu and Mn concentrations remained constant at an average of 0.4 and 0.09 mg per kilogram of fat tissue, respectively, in 200–650 kg bulls.

It can be concluded that the empty-body trace mineral concentration in growing cattle is influenced by allometric growth and the respective stage of the animals' maturity. Muscle tissue comprises the largest share of the animals' bodies (Honig et al., 2022b) and shows increasing Fe and Zn concentrations, which are components of metabolically active proteins. Conversely, increasing bone mineralization and increasing crude fat proportions in fat tissue cause a reduction in the tissues' trace mineral concentrations.

### 3.3 Empty-body trace mineral accretion

**Figure 1:**
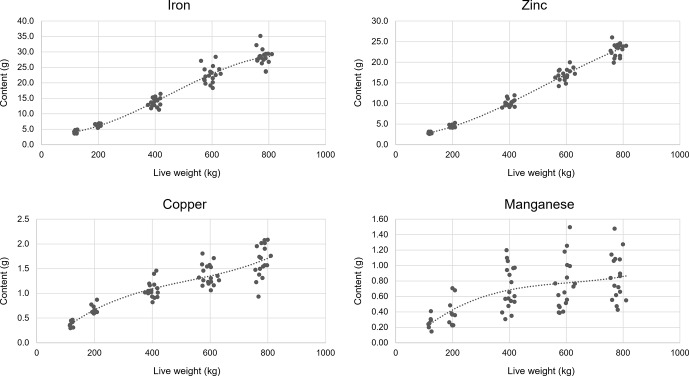
Empty-body trace mineral content in bulls with different live weights.

Third-order polynomial regressions were calculated to determine the empty-body trace mineral content in growing bulls, as illustrated in Fig. 1. A two-way ANOVA showed no significant differences in the residuals of both feed intake groups. Hence, the combined regression equations were used for both groups and are displayed in Table 4.

**Table 4:** Parameters for regression equations on empty-body trace mineral content.

Regression equation: $y=a{\mathrm{LW}}_{i}+b{\mathrm{LW}}_{i}^{2}+c{\mathrm{LW}}_{i}^{3}+e_{i}$					
y	Estimated parameter			SE	$R^{2}$
	a	b	c		
Iron (mg)	22.1630 ± 5.3654	0.0507 ± 0.0194	-0.00004 ± 0.000017	2125.6	0.9467
Zinc (mg)	17.6039 ± 2.8126	0.0256 ± 0.0102	-0.00001 ± 0.000008772	1114.3	0.9779
Copper (mg)	4.1402 ± 0.5249	-0.00478 ± 0.0019	0.000002816 ± 0.000001637	208.0	0.8078
Manganese (mg)	2.6371 ± 0.6768	-0.00288 ± 0.00245	0.000001134 ± 0.000002122	267.4	0.3639

In the regression equation, LW denotes live weight and e is residual error.

**Table 5:** Calculated average trace mineral accretion per kilogram empty-body weight gain (EBWG) in bulls with different live weights.

Empty-body mineral accretion	Live weight								
	100 kg	200 kg	300 kg	400 kg	500 kg	600 kg	700 kg	800 kg	Mean
									100–800 kg
Iron (mg per kg EBWG)	31.103	37.643	41.783	43.523	42.863	39.803	34.343	26.483	37.193
Zinc (mg per kg EBWG)	22.424	26.644	30.264	33.284	35.704	37.524	38.744	39.364	32.994
Copper (mg per kg EBWG)	3.269	2.566	2.033	1.668	1.472	1.445	1.588	1.899	1.992
Manganese (mg per kg EBWG)	2.095	1.621	1.215	0.877	0.608	0.406	0.272	0.206	0.913

Trace mineral accretion rates, in milligrams per kilogram empty-body weight gain (EBWG), were calculated using the first derivative of each regression equation, a method previously applied by Honig et al. (2022a, b). Using regression equations and their derivatives to calculate trace mineral accretion provides the opportunity to calculate typical accretion rates within growing animals. The results can be used to adjust feeding recommendations to fit the growing Fleckvieh bulls' trace mineral requirements.

The accretion rates of the trace minerals Fe, Zn, Cu, and Mn in growing bulls are presented in Table 5. Zn accretion per kilogram EBWG increases in growing bulls, which can be attributed to muscle growth and the increasing Zn concentration in muscle tissue (Table 3). Accretion rates of Mn decrease during growth, which is an effect of decreasing Mn concentrations in hide, fat, tendon, and bone tissues of growing bulls. Fe accretion attained a maximum at 400 kg live weight and declined afterwards. Cu accretion showed a decline until 600 kg live weight and increased slightly afterwards. An increase in Cu accretion in animals with a high live weight may be associated with increasing Cu accumulation in the liver. The liver serves as copper storage in the animal's body and maintains Cu homeostasis until surplus Cu can be excreted via the bile. Peak Fe accretion in 400 kg bulls is attributable to a high Fe concentration and accretion in the blood and organs fraction.

Previous research into trace mineral accretion determined the accretion rates of Fe, Zn, Cu, and Mn to range between 41.4 and 51.5, between 26.4 and 51.4, between 2.0 and 3.3, and between 1.5 and 2.7 mg per kilogram EBWG, respectively, in Fleckvieh bulls with a 200–650 kg live weight fed ad libitum (Kirchgessner et al., 1994b). Hence, accretion rates were reported to increase. However, our study shows more variable accretion patterns for the individual trace minerals, especially for Fe. The total amount of trace mineral accretion depends on the animals' daily weight gain, which was not considered in Table 5.

Increasing trace mineral accretion rates in growing Fleckvieh bulls are associated with increasing mineral requirements, as specified by GfE (1995). In Germany, mean recommendations for dietary Fe, Cu, Zn, and Mn concentrations are 50, 9, 40, and 40 mg per kilogram DM intake, respectively, for fattening cattle from a 175 kg live weight (GfE, 1995). The requirements reported by NRC (2016) for Fe, Cu, Zn, and Mn in beef cattle diets are 50, 10, 30, and 20 mg per kilogram DM intake, respectively. The dietary Cu supply recommended by GfE (1995) and NRC (2016) is in line with observations by Costa e Silva et al. (2015), who reported dietary Cu requirements to be 9.5 mg per kilogram DM intake. Furthermore, Costa e Silva et al. (2015) stated that dietary Fe, Zn, and Mn requirements are 218, 61, and 9.6 mg per kilogram DM intake, respectively, which widely differs from the GfE and NRC recommendations.

Differences in the dietary trace mineral supply recommendations can be attributed to differing trace mineral assessment and calculation methods. In our study, the trace mineral supply per kilogram DM exceeded the requirements recommended by GfE (1995). Furthermore, HE-fed bulls showed significantly higher trace mineral intake than NE-fed bulls, as illustrated in Table 2. The trace mineral supply could have covered higher trace mineral accretion, but physiological homeostasis regulates the amounts of trace minerals in the animals' body. Excessive trace mineral intake results in excretion, not accretion, in body tissues. Therefore, an impairment of the animals' body function is not expected, but higher excretion rates may be related to increased soil pollution. Hence, data covering trace mineral concentration and accretion in bulls' body tissues and empty bodies correspond to the animals' physiological growth process and can be used to derive the net trace mineral requirements of growing bulls in their respective stages of maturity.

Different trace mineral concentrations and accretion rates are expected in other cattle breeds if their body composition deviates from the present animals. Von Soosten et al. (2023) indicated lower protein but higher energy and phosphorus accretion in growing Holstein bulls, which showed higher body fat and ash concentrations in high final weights.

## 4 Conclusion

The empty-body trace mineral concentration is influenced by the allometric growth of individual body tissue fractions and changes related to the animals' respective stage of maturity. The trace minerals Fe and Zn show the highest empty-body trace mineral concentrations in cattle. Growing Fleckvieh bulls' bodies exhibited increasing Zn but decreasing Cu and Mn proportions. The empty-body Fe concentration was comparable in bulls with 120 and 780 kg live weights.

Zn accretion increased while Mn accretion declined steadily during cattle growth. Peak Fe accretion was observed in bulls with a 400 kg live weight, whereas animals with a 600 kg live weight exhibited the lowest Cu accretion rates. Feeding high-concentrate rations did not alter the body trace mineral concentration nor mineral accretion per kilogram EBWG.

A comparison of trace mineral accretion in previous and present Fleckvieh bulls shows more variable accretion patterns for the individual trace minerals in present bulls. Furthermore, the total trace mineral accretion depends on the animals' daily weight gain, which was increased by HE feeding during certain stages of the fattening period. Hence, the provided data on trace mineral accretion can be used to reassess and, if necessary, adjust the feeding recommendations with respect to the trace mineral requirements of growing Fleckvieh bulls. The mean trace mineral accretion rates in growing bulls can also be applied to adjust the trace mineral balance and excretion calculations with respect to the current practical conditions in German fattening bull farms.

## Data Availability

Data are available from the corresponding author upon reasonable request.

## References

[bib1.bib1] Abramowicz B, Kurek Ł, Chałabis-Mazurek A, Lutnicki K (2021). Copper and iron deficiency in dairy cattle. J Elementol.

[bib1.bib2] Cabrera MC, Ramos A, Saadoun A, Brito G (2010). Selenium, copper, zinc, iron and manganese content of seven meat cuts from Hereford and Braford steers fed pasture in Uruguay. Meat Sci.

[bib1.bib3] Cerone SI, Sansinanea AS, Streitenberger SA, Garcia MC, Auza NJ (1998). The effect of copper deficiency on the peripheral blood cells of cattle. Vet Res Commun.

[bib1.bib4] Costa e Silva LF, de Campos Valadares Filho S, Engle TE, Rotta PP, Marcondes MI, Silva FAS, Martins EC, Tokunaga AT (2015). Macrominerals and trace element requirements for beef cattle. PLoS ONE.

[bib1.bib5] Council Regulation (EC) No 1099/2009 (2009). Of 24 September 2009 on the protection of animals at the time of killing. Official Journal of the European Union.

[bib1.bib6] Directive 2010/63/EU (2010). Of 22 September 2010 on the protection of animals used for scientific purposes. Official Journal of the European Union.

[bib1.bib7] Domaradzki P, Florek M, Staszowska A, Litwińczuk Z (2016). Evaluation of the mineral concentration in beef from Polish native cattle. Biol Trace Elem Res.

[bib1.bib8] (1995). Energie- und Nährstoffbedarf landwirtschaftlicher Nutztiere, No 6, Empfehlungen zur Energie- und Nährstoffversorgung der Mastrinder.

[bib1.bib9] Giuffrida-Mendoza M, Arenas de Moreno L, Uzcátegui-Bracho S, Rincón-Villalobos G, Huerta-Leidenz N (2007). Mineral content of longissimus dorsi thoracis from water buffalo and Zebu-influenced cattle at four comparative ages. Meat Sci.

[bib1.bib10] Honig AC, Inhuber V, Spiekers H, Windisch W, Götz K-U, Ettle T (2020). Influence of dietary energy concentration and body weight at slaughter on carcass tissue composition and beef cuts of modern type Fleckvieh (German Simmental) bulls. Meat Sci.

[bib1.bib11] Honig AC, Inhuber V, Spiekers H, Windisch W, Götz K-U, Strauß G, Ettle T (2022). Content and gain of macro minerals in the empty body and body tissues of growing bulls. Meat Sci.

[bib1.bib12] Honig AC, Inhuber V, Spiekers H, Windisch W, Götz K-U, Schuster M, Ettle T (2022). Body composition and composition of gain of growing beef bulls fed rations with varying energy concentrations. Meat Sci.

[bib1.bib13] Kirchgeßner M (2004). Tierernährung, 11th neu überarbeitete Auflage.

[bib1.bib14] Kirchgessner M, Schwarz FJ, Reimann W, Heindl U, Otto R (1994). Deposition of energy and nutrients and utilization of energy for growth in fattening cattle (German Simmental). J Anim Physiol An N.

[bib1.bib15] Kirchgessner M, Heindl U, Schwarz FJ (1994). Content and deposition of trace elements in various tissues and in the empty body of growing German Simmental bulls. J Anim Physiol An N.

[bib1.bib16] Machen M, Montgomery T, Holland R, Braselton E, Dunstan R, Brewer G, Yuzbasiyan-Gurkan V (1996). Bovine hereditary zinc deficiency: lethal trait A 46. J Vet Diagn Invest.

[bib1.bib17] Mateescu RG, Garmyn AJ, Tait Jr RG, Duan Q, Liu Q, Mayes MS, Garrick DJ, Van Eenennaam AL, VanOverbeke DL, Hilton GG, Beitz DC, Reecy JM (2013). Genetic parameters for concentrations of minerals in longissimus muscle and their associations with palatability traits in Angus cattle. J Anim Sci.

[bib1.bib18] Mohri M, Poorsina S, Sedaghat R (2010). Effects of parenteral supply of iron on RBC parameters, performance, and health in neonatal dairy calves. Biol Trace Elem Res.

[bib1.bib19] NRC (2016). National Academies of Sciences, Engineering, and Medicine.

[bib1.bib20] Ramos A, Cabrera MC, Saadoun A (2012). Bioaccessibility of Se, Cu, Zn, Mn and Fe, and heme iron content in unaged and aged meat of Hereford and Braford steers fed pasture. Meat Sci.

[bib1.bib21] Schäffer E (2021). Leistungsprüfung und Beratung in der Milchviehhaltung in Bayern 2021.

[bib1.bib22] Schäffer E (2022). Veredelung Fleisch/Fleischleistungsprüfung in Bayern.

[bib1.bib23] Schwarz FJ, Heindl U, Kirchgessner M (1995). Content and deposition of major mineral elements in different tissues and empty body of growing bulls of the German Simmental breed. Arch Anim Nutr.

[bib1.bib24] Somogyi T, Holló I, Csapó J, Anton I, Holló G (2015). Mineral content of three several muscles from six cattle genotypes. Acta Aliment.

[bib1.bib25] Staley GP, van der Lugt JJ, Axsel G, Loock AH (1994). Congenital skeletal malformations in Holstein calves associated with putative manganese deficiency. J S Afr vet Ass.

[bib1.bib26] VDLUFA (2012). Bd III Die chemische Untersuchung von Futtermitteln.

[bib1.bib27] Von Soosten D, Meyer U, Dänicke S (2023). Forum angewandte Forschung in der Rinder- und Schweinefütterung.

[bib1.bib28] Wysocka D, Snarska A, Sobiech P (2020). Iron in cattle health. J Elementol.

